# Sugammadex and Acceleromyography Used During a Lensectomy in a Sea Lion (*Zalophus californianus*)

**DOI:** 10.3390/ani15192831

**Published:** 2025-09-28

**Authors:** Magdalena Nowak, Shawn Johnson, Claire Simeone, Rocio Canales, Eduardo Huguet-Baudin, Martina Mosing

**Affiliations:** 1 Department of Clinical Diagnostics and Services, Vetsuisse Faculty, University of Zurich, 8057 Zurich, Switzerland; magdalena.nowak@uzh.ch; 2 Anaesthesiology and Perioperative Intensive-Care, Clinical Centre for Small Animal Health and Research, Clinical Department for Small Animals and Horses, University of Veterinary Medicine Vienna, 1210 Vienna, Austria; 3 Sea Change Health, Sunnyvale, CA 94089, USA; shawn@seachangehealth.org (S.J.); claire@seachangehealth.org (C.S.); 4 Veterinary Department, Mundomar Benidorm, 03503 Benidorm, Spain; 5 Oftalmovet, 46023 Valencia, Spain; oftalmovet2@gmail.com

**Keywords:** aceleromyography, California sea lion, cataracts, clinical safety, marine mammal anesthesia, neuromuscular monitoring, pinnipeds, reversal of neuromuscular blockade, rocuronium, sugammadex, train-of-four

## Abstract

Neuromuscular blocking agents (NMBAs) are essential in intraocular surgeries to improve surgical conditions and ensure optimal ventilation. However, residual blockade can pose significant risks, particularly in pinnipeds due to their unique diving physiology. This case report describes the use of sugammadex for reversing rocuronium and AMG for monitoring neuromuscular block (NMB) in a California sea lion undergoing lensectomy. The objective is to evaluate the feasibility and safety of sugammadex for reversal of rocuronium-induced neuromuscular blockade and acceleromyography (AMG) for monitoring neuromuscular function in pinnipeds, with the goal of improving anesthetic management and recovery. Rocuronium (0.3 mg/kg IV) was used to achieve complete NMB, and an additional 0.1 mg/kg IV was administered to prolong the block. Sugammadex (1 mg/kg IV) reversed the NMB, with recovery within 90 s. Neuromuscular function was monitored using AMG, with the ulnar nerve of the foreflipper as the stimulation site. AMG allowed for an objective assessment of neuromuscular function, ensuring accurate titration of the NMBA and reversal agent. This is the first report documenting the use of sugammadex for the reversal of rocuronium and AMG for neuromuscular monitoring in a sea lion. This successful application highlights the potential of these techniques to improve anesthesia protocols, patient safety, and welfare in marine mammal medicine.

## Simple Summary

Eye surgery is a relatively common procedure in older sea lions in captivity. In this case, a sea lion underwent intraocular surgery to remove a bilateral cataract. A neuromuscular blocking agent (rocuronium) was given to guarantee a central eye position during surgery, and at the end of surgery, a reversal agent (sugammadex) was used for the first time in this species to quickly reverse the neuromuscular block (NMB), allowing for a controlled and prompt recovery. To guide the NMB, a monitoring modality (acceleromyography) was used to measure the depth of the NMB and ensure precise timing of drug administration. This technique has not been described in marine mammals yet. The sea lion recovered without complications. This report documents the first use of two veterinary techniques—acceleromyography to monitor muscle relaxation and sugammadex to reverse it—in a California sea lion. These methods proved feasible and safe, and they show potential to improve anesthesia safety, recovery, and overall welfare in pinnipeds undergoing medical procedures.

## 1. Introduction

California sea lions (*Zalophus californianus*) are long-lived pinnipeds, with maximum lifespans of ~19 years for males and 25 years for females in the wild, and up to ~30 years in managed care [1,2]. They exhibit marked sexual dimorphism: adult males typically measure 2.0–2.5 m in length and weigh 200–400 kg, while adult females average 1.5–2.0 m and 50–110 kg [3,4]. Ocular diseases are a major welfare concern in pinnipeds under human care, with prevalence ranging from 22% to over 40% in large surveys and up to 80% in some reports, compared to animals living in the wild [5,6,7]. Frequently observed conditions include cataracts, keratitis, corneal opacity, and lens disorders. These often progress to visual impairment, which in captive pinnipeds has been associated with reduced ability to track or capture prey, hesitancy or disorientation when navigating enclosures, and changes in social behavior (e.g., less interaction with conspecifics). Such impairments thereby compromise feeding, social behavior, and environmental navigation, ultimately reducing welfare [8]. As a result, tsurgical interventions are often required to restore visionand improve quality of life. However, these procedures require precise eye positioning and complete immobility, as even minor movements can compromise surgical outcomes [9].

Their high incidence has been linked to multiple factors such as water quality (freshwater and chlorine exposure), high oxidation–reduction potential from disinfection, ultraviolet light exposure from reflective pool environments, as well as age and social or behavioral influences [5]. In California sea lions, advanced ocular disorders like premature cataracts and lens luxations pose particular surgical challenges, where neuromuscular blocking agents (NMBAs) are critical to achieve central eye positioning, optimal surgical conditions, reduced intraocular pressure, and improved ventilation control [10]. Non-depolarizing neuromuscular blocking agents (NMBAs) act at the neuromuscular junction by competitively binding to nicotinic acetylcholine receptors on the motor endplate, thereby preventing acetylcholine from triggering depolarization and subsequent muscle contraction. This pharmacological blockade produces temporary skeletal muscle paralysis [11,12]. The block of extraocular muscles causes central eye position and abolishes every palpebral reflex–optimal surgical conditions for intraocular surgery.

Rocuronium is a non-depolarizing NMBA of the aminosteroidal class. In veterinary species, rocuronium shows a rapid onset (time from administration to disappearance of twitches from neuromuscular stimulation) and intermediate duration of action (20–40 min) [13,14]. In dogs, an intravenous dose of 0.4 mg/kg produces neuromuscular blockade within 98 ± 52 s, lasting for approximately 32.3 ± 8.2 min [15]. In cats, a dose of 0.6 mg/kg IV results in an onset time of 46 ± 11 s and a shorter duration of 13.2 ± 2.7 min [16]. In horses, 0.3 mg/kg IV leads to an onset of 2.3 ± 2 min and a duration of 32 ± 18.6 min [17]. Rocuronium produces minimal cardiovascular effects with no clinically relevant changes in heart rate [18]. A slight and transient increase in heart rate has occasionally been observed in humans and dogs [18,19]. Rocuronium does not cause histamine release, and has minimal vagolytic activity [20,21]. The risk of residual neuromuscular blockade (NMB) in pinnipeds poses a significant concern. Their unique respiratory physiology—including voluntary apnea and a pronounced diving reflex—requires a smooth, quick and complete recovery from anesthesia [22,23]. Sugammadex, a modified γ-cyclodextrin, provides rapid and predictable reversal of rocuronium-induced blockade without the need for anticholinergics in small and large animals [24,25,26]. Its ability to prevent residual paralysis makes it particularly valuable in high-risk species [13,15,16]. By ensuring rapid and complete reversal, sugammadex not only facilitates safe extubation and prevents aspiration, but also supports postoperative welfare through a faster return to normal behaviors.

Monitoring neuromuscular function is essential for the safe and effective use of neuromuscular blocking agents (NMBAs) and their reversal agents. Acceleromyography (AMG) is an objective, quantitative, real-time method of neuromuscular monitoring that evaluates the degree of neuromuscular transmission by measuring the acceleration of muscle contractions in response to peripheral nerve stimulation. It relies on the principle that the peak acceleration of a limb following nerve stimulation correlates directly with the force generated by the corresponding muscle contraction following the second law of Newton [27]. The equipment typically consists of two electrodes where one is placed over a peripheral nerve and another serving as a reference (zero) electrode, a stimulator to deliver electrical impulses in milliampers (mA), and an accelerometer attached to the corresponding muscle or digit to detect movement [28]. Compared with subjective visual or tactile methods (e.g., peripheral nerve stimulator with palpation), AMG provides greater precision, sensitivity, and objectivity for detecting partial neuromuscular blockade. It is most commonly applied to evaluate the train-of-four (TOF) ratio, single twitch, tetanic stimulation, and post-tetanic count, all of which provide information on the depth of the block and adequacy of recovery. In veterinary medicine, AMG has been successfully used in dogs, cats, and horses to guide NMBA dosing and assess recovery [16,17,24]. Despite this, its use has not previously been reported in pinniped species.

To facilitate its application in these animals, one potential stimulation site is the ulnar nerve, which arises from the brachial plexus in pinnipeds, formed by the sixth cervical to the first thoracic spinal nerves [29,30]. It runs along the medial aspect of the forelimb, passing posterior to the olecranon process, and provides motor and sensory innervation to the musculature and soft tissues [29,30]. Due to the paddle-like structure of pinniped fore flippers, the ulnar nerve remains deep, but can be accessed near the ulnar nerve groove, just medial to the olecranon making it suitable for nerve stimulation and neuromuscular monitoring. The use of rocuronium and sugammadex in pinnipeds has been presented in a recent conference abstract [31], but no peer-reviewed accounts are currently available. To our knowledge, this case therefore represents the first published description of sugammadex use in a pinniped, and uniquely, the first report of acceleromyography for neuromuscular monitoring in this taxon.

The aim of this case report is to describe the first use of sugammadex for rocuronium reversal and acceleromyography for neuromuscular monitoring in a California sea lion undergoing lensectomy. The lack of published information on the use of acceleromyography in pinnipeds highlights the novelty of this report, which provides the first description of this technique in combination with sugammadex. The objective is to evaluate the feasibility and safety of sugammadex for reversal of rocuronium-induced neuromuscular blockade and acceleromyography for monitoring neuromuscular function in pinnipeds, with the goal of improving anesthetic management and recovery.

## 2. Case Presentation

### 2.1. Medical History

A 19-year-old male California sea lion (*Zalophus californianus*), weighing 187 kg, housed at Mundomar Animal Park in Benidorm, Spain, in a social group of conspecifics, was scheduled for ophthalmic surgery due to bilateral hypermature cataracts. Other sea lions within the same group had also been noted to present with similar ocular conditions. Before the procedure, the sea lion showed signs consistent with visual impairment, including impaired visually tracking of fish during feeding sessions and hesitancy when navigating his enclosure. One week prior to surgery, topical ophthalmic treatment was initiated to optimize ocular conditions for the procedure. The regimen included tri-antibiotic drops containing gramicidin, neomycin sulfate, and polymyxin B sulfate (Oftalmowell^®^, Teofarma S.R.L., Pavia, Italy), administered as one drop three times daily (TID) in both eyes (OU); ciprofloxacin 3 mg/mL (Oftacilox^®^, NTC S.R.L., Milano, Italy), one drop TID OU; prednisolone 10 mg/mL (Pred Forte^®^, AbbVie Spain, S.L.U., Madrid, Spain), one drop TID OU; and nepafenac 3 mg/mL (Nevanac^®^, Novartis Europharm Limited, Dublin, Irland), one drop twice daily (BID) OU.

Systemic preoperative treatment, initiated 72 h before the procedure, consisted of oral gabapentin (Gabapentina, Kern Pharma, Barcelona, Spain,) at 800 mg BID, prednisone (Prednisona Cinfa, Laboratorios Cinfa, Huarte, Spain) at 75 mg once daily, and famotidine (Famotidina Cinfa, Laboratorios Cinfa, Huarte, Spain) at 80 mg BID. Blood work performed under anesthesia revealed values within normal limits.

### 2.2. Anaesthetic Management

The animal was premedicated with midazolam (Midazolam Normon, Normon, Spain) (0.2 mg/kg Intramuscular (IM), butorphanol (Butomidor, Richter Pharma, Wels, Austria) (0.2 mg/kg IM), medetomidine (Domtor, Ecuphar, Spain) (0.01 mg/kg IM), and ketamine (Ketamidor, VetViva Richter, Wels, Austria) (1 mg/kg IM), administered into gluteus maximus muscle. No additional induction agents were necessary for endotracheal intubation using a 18 mm internal diameter (ID) endotracheal tube (Surgivet, ICU Medical, San Clemente, CA, USA). Anaesthesia was maintained using isoflurane (Isoflutek, Laboratorios Karizoo, Caldes de Montbui, Spain) in oxygen delivered via a circle system MDS Matrx VML Anesthesia Machine (MDS Inc., Versailles, OH, USA). An 18-gauge IV catheter was placed after intubation using ultrasound guidance. Initial fresh gas flow was set to 2–3 L/min and reduced to 0.6–1 L/min during maintenance. Ventilation was manually controlled with a peak inspiratory pressure (PIP) of approximately 12 cmH_2_O and positive end-expiratory pressure (PEEP) of approximately 8 cmH_2_O, adjusted via the APL valve, resulting in a tidal volume of ~900 mL and a respiratory rate of 10–15/min. Depth of anesthesia was assessed by the anesthesiologist based on palpebral and withdrawal reflexes (flipper pinch), jaw and muscle tone, and cardiovascular responses (e.g., changes in heart rate). After administration of the NMBA, monitoring of anesthetic depth relied primarily on cardiovascular parameters. Hypothermia prevention was ensured using two thermal blankets (Cecotec, Alfafar, Valencia, Spain), one placed on top and one underneath the animal.

Monitoring consisted of side stream capnography, pulse oximetry (SpO_2_ probe placed on the tongue), electrocardiography (heart rate and arrhythmia analysis), and non-invasive blood pressure measurement, using a multi parameter monitor (Mindray PM 9000 Vet, Mindray Animal Medical Technology North America, Mahwah, NJ, USA). Internal body temperature was measured rectally (BioTex Temp Loop, BOSCOGEN, Irvine, CA, USA). Inspired and expired concentrations of oxygen and isoflurane were measured using a multi gas analyzer (Vamos, Dräger, Lübeck, Germany). All data was manually recorded every 5 min (Table S1). Cefazolin (22 mg/kg, Cefazolina Normon, Tres Cantos, Spain) was administered intravenously (IV) into the jugular vein 20 min before the start of surgery. Additionally, a saline infusion (Fisiovet, Braun, Rubi, Spain) was administered IV throughout the procedure at a rate of 5 mL/kg/h, as lactate-containing solutions are generally avoided in marine mammal anesthesia due to concerns about elevated lactate levels [32].

### 2.3. Nerve Stimulation, Neuromuscular Blocking and Monitoring

**Figure 1:**
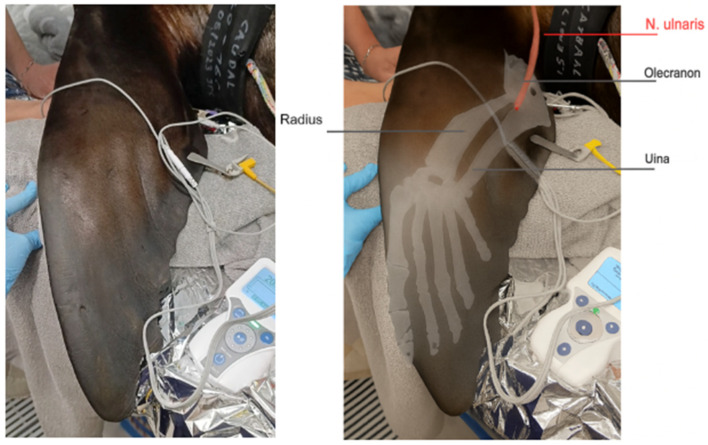
Anatomical landmarks and electrode positioning for ulnar nerve stimulation in the foreflipper of a California sea lion. Figure 1 is adapted that the bony anatomy was adapted from Zhao et al. (2022) [33], and AI was used only for aesthetic refinement (OpenAI via ChatGPT o4-mini).

For neuromuscular blockade monitoring, the ulnar nerve was selected for stimulation using a train-of-four (TOF) stimulation pattern (Stimpod, Xavant, Pretoria, South Africa). The sea lion was positioned in lateral recumbency with the fore flipper extended and supported to allow for free movement of the limb. A transcutaneous electrical nerve mapping probe (Stimpod, Xavant, Pretoria, South Africa) was used to locate the optimal stimulation site. The probe was applied firmly to the skin at the medial aspect of the fore flipper, near the olecranon process, and moved slowly along the inner surface of the fore flipper. The position where the current of 20 mA with stimulation of 5 Hz induced maximal movement of the flipper was identified. Once the optimal position for the stimulation of the ulnar nerve was located, the stimulating electrode using a stainless steel luer-lock needle was placed through the skin at this precise site. The zero electrode was placed two centimeters proximal to the stimulation electrode on the dorsolateral surface of the antebrachial region (Figure 1).

**Figure 2:**
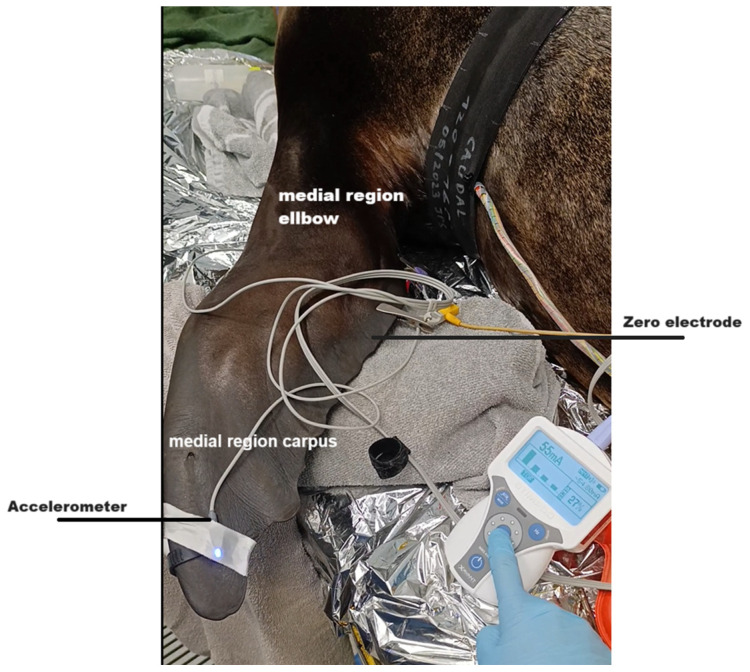
Placement of Acceleromyography (AMG) Transducer for Neuromuscular Monitoring in the Fore Flipper of a California Sea Lion. Figure 2 is adapted that the bony anatomy was adapted from Zhao et al. (2022) [33], and AI was used only for aesthetic refinement (OpenAI via ChatGPT o4-mini).

For AMG, the transducer was positioned on the dorsal side of the fore flipper, caudally, at the site of maximal movement (Figure 2) and secured with tape. The stimulation site was localized using a nerve mapping probe (20 mA at 5 Hz). Neuromuscular monitoring was then performed with a TOF pattern (4 stimuli, 2 Hz, pulse width ~0.2 ms, repeated every 15 s; supramaximal current 50 mA), allowing fpr the continuous assessment of neuromuscular blockade and accurate evaluation of muscle responses during anaesthesia.

AMG values were normalized to a baseline TOF ratio of 1.0 prior to NMBA administration to minimize the risk of misinterpretation due to overshoot, which have occasionally been reported with AMG [34]. The monitoring was continued for a period of 10 min to allow for stabilization of the recordings before the administration of the NMBA.

Deep NMB was achieved using rocuronium (Rocuronium Kabi, Fresenius Kabi, Bad Homburg von der Höhe, Germany) at a dose of 0.3 mg/kg IV for surgery of the first eye. Complete blockade (TOF count 0/4) was reached within 4 min. The first twitch returned 47 min after administration, followed by the second twitch at 51 min. Because the surgeon did not report any changes in eye position, a second dose of rocuronium (0.1 mg/kg IV) was administered only 83 min after the initial dose for the surgery on the second eye, when the TOF ratio was 31% and TOF count 4/4. Two minutes after the second dose, the TOF ratio was 35% with a TOF count of 4/4. After another 28 s, the TOF count decreased to 1/4 and remained at that level until deep NMB was achieved. Complete NMB, with no visible twitches, was achieved at 4 min and 48 s after re-dosing. The first twitch returned 37 min after the second dose, the second at 46 min. After 48 min a TOF ratio of 9% was visible. Sugammadex (Sugammadex, Fresenius Kabi, Bad Homburg von der Höhe, Germany) was administered at a dose of 1 mg/kg IV, 53 min after the second rocuronium dose, still at a TOF ratio of 9%. Following sugammadex administration, the TOF ratio increased from 0.09 to 0.90 within 54 s and reached 1.15 after 90 s. The transient overshoot above 1.0 is a known phenomenon with AMG monitoring and has been reported previously despite normalization to baseline. No signs of recurarization were observed during the subsequent three minutes of monitoring; however, continuous monitoring for at least 10–15 min is generally recommended to reliably exclude recurrence of neuromuscular block, and the short monitoring period in this case represents a limitation.

### 2.4. Recovery and Postoperative Management

Recovery was smooth, with extubation performed 20 min after cessation of isoflurane. The animal assumed sternal recumbency immediately after extubation and was able to stand and walk 30 min after discontinuation of isoflurane. The animal was closely monitored during recovery, with administration of reversal drugs IM into gluteus maximus muscle: atipamezole (Antisedan, Ecuphar, Brussels, Belgium) (0.075 mg/kg IM), flumazenil (Flumazenil, B. Braun, Melsungen, Germany) (0.015 mg/kg IM), and naloxone (Naloxona, Kern Pharma, Terrassa, Spain) (0.01 mg/kg IM). Other postoperative medications included dexamethasone (Dexadreson, MSD Animal Health, Boxmeer, The Netherlands) (0.16 mg/kg IM), cefovecin (Convenia, Zoetis, Louvain-la-Neuve, Belgium) (4 mg/kg SQ), enrofloxacin (Baytril, Elanco, Leverkusen, Germany) (5 mg/kg SQ), per ophthalmology protocol, and others as outlined.

Following recovery and subsequent ophthalmic healing, improved orientation and feeding behavior were reported, suggesting a welfare benefit from restored vision.

## 3. Discussion

This case provides the first documented evidence that sugammadex and acceleromyography (AMG) can be successfully applied in a California sea lion (*Zalophus californianus*) undergoing ophthalmic surgery to manage and reverse neuromuscular blockade (NMB). The use of sugammadex for reversing rocuronium-induced blockade has been well documented in veterinary species. Reported doses range from 0.5 mg/kg in horses to 4–5.5 mg/kg in dogs and ponies, with reversal typically occurring within 2–4 min [24,25,35,36]. Our case demonstrates that even a relatively low dose of sugammadex (1 mg/kg, administered at a TOF ratio of 0.09 indicating deep-to-moderate block) can provide complete and predictable reversal in pinnipeds. The choice of this low dose was guided by AMG monitoring and clinical context, aiming to balance efficacy with cost-effectiveness. However, dosing must be individualized until species-specific pharmacokinetic and pharmacodynamic data become available, and objective neuromuscular monitoring remains essential. Rapid recovery minimized the risks associated with extubation. In comparison, traditional reversal agents such as neostigmine are less reliable and require anticholinergic co-administration, which may cause cardiovascular and secretory side effects [37]. Sugammadex directly encapsulates rocuronium and has demonstrated superior efficacy and safety in dogs, horses, and ponies [24,25,35,36]. Our findings suggest that these benefits may extend to pinnipeds as well. Importantly, AMG guided the decision to administer this reduced dose of sugammadex (1 mg/kg). This individualized titration, based on neuromuscular function rather than fixed dosing, ensured both safety and efficacy while minimizing drug use. Reported doses in other species are typically higher, with 2–4 mg/kg recommended in dogs and humans for moderate to deep block [13,36,38]. The fact that complete and predictable reversal was achieved in our case with a lower dose suggests potential interspecies differences in response to sugammadex. Considering the high cost of this drug, such an individualized, lower-dose approach may be particularly relevant in veterinary settings where financial constraints often limit access to advanced pharmaceuticals. Thus, AMG contributed not only to clinical safety but also to cost-effective anesthetic management. Our findings are consistent with a recent conference case series where sugammadex was also successfully used to reverse rocuronium in pinnipeds. In that series, however, one case experienced prolonged recovery, most likely because a comparatively larger dose of rocuronium had been administered without sufficient reversal [31]. This observation further underscores the importance of individualized dosing strategies and highlights the potential benefit of quantitative monitoring such as AMG to avoid delayed recoveries. AMG was also technically feasible in this pinniped and clinically beneficial for optimizing both the depth and timing of NMB use and reversal. In pinnipeds, the bones of the forelimbs and hindlimbs are shortened and encased in a thick layer of blubber and skin, forming flippers. Digits are elongated and webbed, providing propulsion in water, while joints have a reduced range of motion compared to terrestrial mammals [39]. Despite anatomical challenges—such as the modified forelimb structure and the deep location of the ulnar nerve—a transcutaneous nerve mapping probe enabled accurate identification of the stimulation site [22,23]. Once positioned, the AMG transducer reliably recorded TOF responses throughout anesthesia, demonstrating that neuromuscular monitoring techniques commonly used in terrestrial species can be successfully adapted to marine mammals. Moreover, the use of AMG allowed individualized titration of rocuronium, confirming deep NMB (TOF 0/4) within 4 min after a 0.3 mg/kg IV dose, which is a longer onset time but lower dose to achieve deep block than described for other land mammals. In dogs, the onset is reported as 98 ± 52 s with 0.4 mg/kg IV, in cats 46 ± 11 s with 0.6 mg/kg IV, and in horses 2.3 ± 2 min with 0.3 mg/kg IV [15,16,17]. It also revealed a recovery pattern that differed from domestic species. The return of the first twitch occurred 47 min after the initial 0.3 mg/kg IV dose and 48 min after a supplemental 0.1 mg/kg dose, despite a relatively low cumulative dose. In contrast, 0.3 mg/kg rocuronium typically results in a block duration of 20–35 min in dogs and horses, and 10–15 min in cats [15,16,17,35]. Interestingly, a transient increase in TOF ratio from 31% to 35% was observed two minutes after the second rocuronium dose, despite the expected progression of neuromuscular block. This was followed by a gradual decrease in TOF count over the next several minutes, with complete NMB achieved only after 4 min and 48 s. The sea lion’s prolonged and delayed response to rocuronium suggests that pinnipeds may metabolize the drug more slowly due to species-specific hepatic clearance, reduced protein binding, or slower elimination [40,41]. These findings underscore the importance of objective quantitative neuromuscular monitoring to ensure both optimal surgical conditions (onset and depth of block) and safe recovery from NMB. Furthermore, AMG proved instrumental not only for ensuring adequate blockade during surgery but also for guiding the recovery phase. It verified complete NMB intraoperatively and enabled real-time monitoring of twitch return and TOF ratio during recovery, allowing for the precise dosing of the reversal agent. By detecting a TOF ratio of only 9% at 53 min after the second dose, AMG enabled timely and precise reversal. A transient TOF ratio overshoot (>1.0) was also observed—a phenomenon well recognized in acceleromyography, often due to the staircase effect and normalization against baseline values, rather than reflecting true neuromuscular recovery [42]. Because twitch amplitudes may increase or decrease over time due to these effects, clinicians could misinterpret such changes as recovery from, or progression of, neuromuscular blockade, even when the drug effect has not actually changed. Awareness of this artifact is essential for consistent interpretation of AMG values and underscores the need for prolonged monitoring to exclude residual blockade and recurarization. Without such monitoring, residual blockade could have gone undetected, increasing the risk of hypoventilation, apnea, or respiratory compromise—especially in diving species with voluntary apnea and a strong diving reflex [22,23]. It should also be noted that laryngeal muscle recovery has been shown in both humans and veterinary species to lag behind the recovery of limb muscles [13,26,43]. As full laryngeal function was not verified in our case, we cannot make any definitive statement on this; however, we suggest extubation only several minutes after the return of full limb strength. In clinical anaesthesia, acceleromyography has been widely used to guide extubation, with a TOF ratio ≥ 0.9 generally accepted as the threshold for adequate recovery of airway reflexes in humans [44], and similarly applied in dogs, cats, and horses [16,17,36]. Nevertheless, veterinary studies in ponies indicate that laryngeal function may still recover more slowly than limb muscles [25], raising concerns about relying solely on limb-based AMG monitoring. In pinnipeds, no validated criteria currently exist, but adopting a cautious approach—aiming for a TOF ratio close to 1.0 and delaying extubation briefly after limb twitch recovery—appears prudent until species-specific values are established.

Despite the positive outcome, this case highlights important limitations. There are no species-specific reference values for TOF ratios that indicate adequate spontaneous ventilation in marine mammals, nor standardized guidelines for electrode placement in pinnipeds. This lack of standardization complicates interpretation and raises ethical concerns, as clinicians must rely on extrapolated data from land mammals. Although sugammadex was effective at 1 mg/kg in this case, pharmacokinetic and pharmacodynamic studies are needed to define optimal dosing in pinnipeds. This is especially important in animals with hepatic or renal impairment, given interspecies metabolic variability and the liver-based clearance of rocuronium [37]. Furthermore, due to the high cost of sugammadex, evidence-based, cost-efficient protocols are essential. While no signs of recurarization were observed, the monitoring period was short, which represents a limitation. However, no clinical signs of recurarization were noted during the observation period. Traditionally, after reversal with neostigmine, clinicians delayed extubation for 10–15 min to allow recovery of laryngeal function [45]; in contrast, sugammadex enables earlier extubation, although verification of airway safety remains essential [13]. Future research should aim to establish AMG reference values and electrode positioning guidelines in marine mammals, and to characterize species-specific pharmacokinetics of rocuronium and sugammadex. Finally, developing ethical and clinical standards for NMB monitoring in pinnipeds will be essential to ensure safe and effective anesthesia.

## 4. Conclusions

This case report provides the first documented application of sugammadex and acceleromyography (AMG) in a California sea lion *(Zalophus californianus)* undergoing lensectomy. The successful use of AMG allowed for the objective, real-time monitoring of neuromuscular function, while sugammadex enabled for the rapid and complication-free reversal of rocuronium without any signs of recurarisation. These findings indicate that the use of AMG and sugammadex in pinnipeds is technically feasible and may improve anesthesia safety and recovery. However, as this conclusion is based on a single case, further investigations are necessary to confirm reproducibility, define optimal dosing, and establish species-specific guidelines for neuromuscular monitoring and reversal in marine mammals.

## Data Availability

No new data were created or analyzed in this case report. Anonymized time-stamped TOF/TOFratio traces are provided as Supplementary Material.

## Supplementary Materials

The following supporting information can be downloaded at: https://www.mdpi.com/article/10.3390/ani15192831/s1. Table S1: Vital parameters (SpO_2_, EtCO_2_, HR, MAP, Temp) monitored during anesthesia. SpO_2_ = peripheral oxygen saturation; EtCO_2_ = end-tidal carbon dioxide; HR = heart rate; MAP = mean arterial pressure; Temp = body temperature. Table S2: Train-of-four (TOF) count and ratio traces during anesthesia in a California sea lion.

## Abbreviations

The following abbreviations are used in this manuscript:

AMG	Acceleromyography
BID	Bis in die, twice a day
IM	Intramuscular
IV	Intravenous
NMB	Neuromuscular Block
NMBA	Neuromuscular Blocking Agent
OU	Oculus uterque, both eyes
SQ	Subcutaneous
TOF	Train-of-Four
