# Supplementary material for: Sugammadex and Acceleromyography Used During a Lensectomy in a Sea Lion (Zalophus californianus)
Source: Animals (Basel). 2025 Sep 28;15(19):2831. doi: 10.3390/ani15192831 (PMC12524313; doi:10.3390/ani15192831)
Supplement: Supplementary file 1 [file animals-15-02831-s001.zip › Table S1.pdf]

Table S1. Vital parameters (SpO_2, $EtCO_2$, HR, MAP, Temp) monitored during anesthesia. SpO_2 = peripheral oxygen saturation; $EtCO_2$ = end-tidal carbon dioxide; HR = heart rate; MAP = mean arterial pressure; Temp = body temperature.

Time	SpO_2 (%)	$EtCO_2$ (mmHg)	HR (bpm)	MAP (mmHg)	Temp (°C)
12:00	100	48	50	68	36.0
12:15	100	47	80	69	36.0
12:30	100	45	81	70	36.0
12:45	95	45	82	70	36.0
13:00	100	45	78	65	36.1
Rocuronium 0.3 mg/kg IV					
13:15	100	37	77	68	36.2
13:30	100	39	76	70	36.3
13:45	100	40	75	71	36.5
14:00	100	38	74	67	36.8
14:15	100	37	79	66	36.8
Rocuronium 0.1mg/kg IV					
14:30	100	37	75	55	37
14:45	100	38	75	62	37.2
15:00	100	38	106	67	37.3
15:15	100	37	100	66	37.3
Sugammadex 1mg/kg IV					
15:30	95	36	94	70	37.3

15:45	95	31	120	70	37.3
